# Use of a CME workshop to introduce and promote the specialty of Family Medicine in Ethiopia

**DOI:** 10.4102/phcfm.v2i1.155

**Published:** 2010-08-31

**Authors:** Jane Philpott, Derbew Miliard

**Affiliations:** 1 Department of Family Medicine, Markham Stouffville Hospital, Canada; 2 Department of Family and Community Medicine, University of Toronto, Canada; 3 Department of Surgery, Tikur Anbessa Hospital, Ethiopia; 4 Faculty of Medicine, Addis Ababa University, Ethiopia

**Keywords:** continuing medical education, family practice, internship, medical faculty, residency health personnel

## INTRODUCTION

A growing number of universities in sub-Saharan Africa offer graduate training for generalist physicians and some countries have formally recognised Family Medicine as a specialty.^1,2,3^ Yet, in parts of the region, the concept of Family Medicine is new and not broadly understood.^4^ There is a need for creative and practical ways to introduce policymakers, academics and medical trainees to the specialty of Family Medicine so that its potential benefits may be evaluated and made available.

A groundbreaking continuing medical education (CME) workshop took place in Addis Ababa in March 2009 as an introduction to the specialty of Family Medicine. The event was organised by the Ethiopian Medical Association in collaboration with the University of Toronto’s Department of Family and Community Medicine. The CME format was chosen to present Family Medicine to a broad audience, including senior medical faculty who could lead the implementation of the new specialty, as well as young physicians who could be early recruits to Family Medicine training.

The specialty of Family Medicine has been shown to provide an important contribution to the improvement of health systems worldwide.^5^ Rather than a disease-by-disease approach to health care, Family Medicine focuses on people and populations.^6^ A cadre of well-trained family doctors leading primary health care teams can result in health care delivery that is coordinated, comprehensive and cost-effective.

Currently, the specialty of Family Medicine does not exist in Ethiopia. But its development could be one of the key ways of addressing the overwhelming health concerns of the country. Years ago, the possibility of Family Medicine training in Ethiopia was advocated by Professor Jemal Abdulkadir, an endocrinologist at Addis Ababa University. In 1995, Professor Jemal wrote:

… the place of general practice in Ethiopia’s health care system is still undefined. There are very few incentives to attract young doctors to it as a career. As a result, most see it as a temporary occupation.^7^

Since then, Family Medicine has been adopted as a new specialty by a number of other countries in the region. International partnerships have been encouraged to assist with educational support while Family Medicine is developed as a specialty.^2^

The CME workshop was designed as an interactive forum to introduce a range of stakeholders to this specialty and to consider its benefits for the country. The objectives of the workshop were:

- To investigate the development of the specialty of Family Medicine in Ethiopia.
- To identify appropriate principles for Family Medicine in the Ethiopian context.
- To assess the educational needs in training generalist physicians in Ethiopia.
- To encourage research and writing for publication on the theme of Family Medicine in Ethiopia.
- To outline the next steps toward Family Medicine training, if deemed appropriate.

## METHOD

The CME workshop was organised by the Ethiopian Medical Association in collaboration with the Faculty of Medicine at Addis Ababa University. Facilitators for the workshop included four faculty members from the University of Toronto and one faculty member from Moi University School of Medicine in Kenya. The needs assessment and programme planning took place by means of email dialogue among faculty members from all three locations.

A variety of adult educational methods were used in the workshop to promote reflection and learning by all participants. The programme included formal speeches, didactic lectures, PowerPoint presentations, small-group discussions, role plays, large group interaction and a panel discussion. Informal learning opportunities took place during breaks and mealtime conversations.

The workshop programme included the following session topics:

- Why Family Medicine? – A vision for Family Medicine in Ethiopia.
- Principles of Family Medicine – A global survey.
- Family Medicine in real life – An evolving clinical story with role play.
- Family Medicine in context – Examples from Canada, Brazil and Kenya.
- Clinical scenarios workshop – Applying the principles of Family Medicine in clinical cases.
- Putting it together – Deriving principles for Family Medicine in Ethiopia.
- The way forward – Panel discussion about how to develop Family Medicine as a specialty.

Evaluation of the programme was both formative and summative. Informal feedback was obtained at the end of each session through questions, answers and comments. Participants were asked to complete an evaluation form at the end of each session and a summative evaluation after the closing session.

## RESULTS

The CME workshop was attended by 52 physicians. Twenty-nine of the participants were members of the Addis Ababa University Faculty of Medicine. There were two representatives from Haramaya University in Harar, one from Jimma University in Jimma and one from Mekele University in Tigray. Two physicians were on the executive council of the Ethiopian Medical Association. Seventeen of the participants were general practitioners who work in regional and district hospitals. One representative of the Ministry of Health was in attendance. 

The workshop opened with a session entitled ‘Why Family Medicine?’ that provided an historical and international perspective on the specialty as well as a review of how Family Medicine can contribute to the improvement of health systems.^5^

Then an interactive process was used to develop a preliminary list of roles, skills and values for a Family Medicine specialist in Ethiopia. During Sessions B and D, participants were introduced to various principles of Family Medicine that had been developed in at least three continents. The list of these principles was recorded on a whiteboard. During Session C, ‘Family Medicine in real life’, two faculty members and a facilitator presented a four-scene role play of a doctor–patient interaction throughout a lifetime. Then, in Session E, ‘Clinical scenarios workshop’, participants were divided into three groups, with each group asked to choose a common clinical problem that a generalist physician in Ethiopia would face. The chosen topics were malaria, HIV infection and depression. After a period of preparation each group presented a role play of a doctor–patient interaction based on their chosen clinical problem. Finally, in Session F, ‘Putting it together’, a skilled facilitator worked with the large group to analyse each clinical role play to brainstorm a list of roles, skills and values that were necessary for the physician who might manage such a clinical case. This exercise allowed a simulated study of the work of a potential family physician. The list of characteristics that could define Family Medicine in Ethiopia is shown in Table 1.

**TABLE 1:**
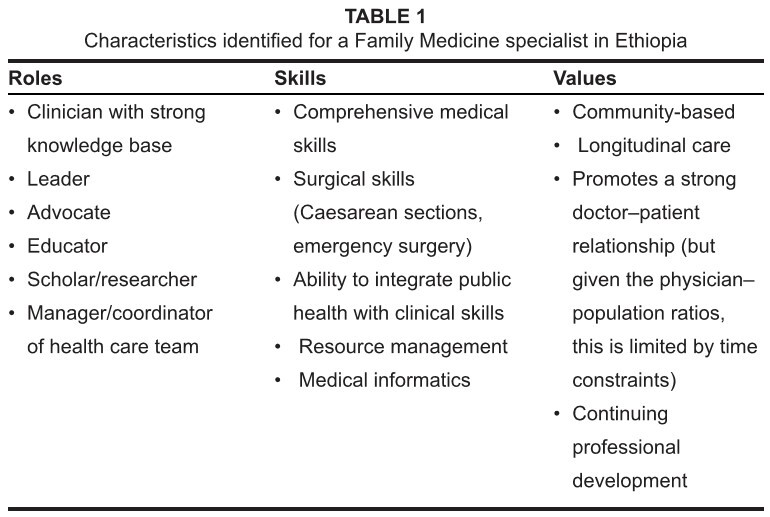
Characteristics identified for a Family Medicine specialist in Ethiopia

Throughout the programme, participants were introduced to the academic literature supporting the development of Family Medicine as a specialty. Facilitators noted that a commitment to research and scholarly writing on the topic of Family Medicine should be a fundamental part of the development of the specialty.

The closing session of the CME involved a panel of key academic leaders. They were asked to consider the next steps that would be necessary to develop the specialty of Family Medicine in Ethiopia. The following key steps were identified:

- Advance the dialogue on the topic of Family Medicine training with the Federal Ministry of Health and the Ministry of Education.
- Develop a Family Medicine advisory committee.
- Establish regular meetings with stakeholders, including the universities and colleges that will supervise the training.
- Begin curriculum development.
- Consider a pilot site to model the Family Medicine team where the first group of residents could be trained.
- Send representatives to the World Organisation of Family Doctors (WONCA) Conference in October 2009 in Rustenburg, South Africa.

Evaluation comments indicated that the workshop was highly successful in terms of exploring the possibility of introducing Family Medicine as a specialty in Ethiopia. Sample comments from the summative evaluation included:

- ‘There is clear evidence that Family Medicine is beneficial in our country.’
- ‘Ethiopia needs Family Medicine to lower health costs and give better health outcomes.’
- ‘Family Medicine can be adjusted based on the context.’
- ‘We ourselves are the ones to start Family Medicine.’
- ‘I want to participate in making this a reality to the best of my ability.’
- ‘Family Medicine in Ethiopia is a necessity!’
- ‘Was very impressive and we hope for better health for Ethiopia.’

## DISCUSSION

Development of the specialty of Family Medicine in Ethiopia could be an important step to improve health outcomes, manage health care costs and distribute more equitable access to care.^8^ Recent reports note the high incidence of health problems in Ethiopia resulting from preventable communicable diseases such as malaria, pneumonia and tuberculosis.^9^ Addressing such circumstances requires a strong system of primary care, including physicians with enhanced training to lead programmes of prevention and treatment before ailments advance to a stage requiring sub-specialty care.

Family Medicine is emerging as a specialty that can offer such enhanced training to physicians. Until recently, the concept of Family Medicine has not been clearly defined in the context of sub-Saharan Africa.^4^ Few resources are available to introduce the specialty to a region where it does not currently exist. This paper describes how a CME programme employed a variety of educational methods to describe the roles, responsibilities and training associated with the specialty of Family Medicine.

The programme clarified the fact that there is not a single definition of Family Medicine that applies to all the countries of the world. Rather, a precise definition of Family Medicine is unique to each local setting and its needs.^10^

Increased awareness of the benefits of developing Family Medicine as a specialty was made possible through didactic sessions and informal discussions. Evaluation results demonstrated an enhanced understanding of how Family Medicine could be defined in Ethiopia and revealed an impressive enthusiasm for the specialty among young physician participants. A creative exercise was successfully used to draft a list of characteristics that could define the roles, skills and values of a Family Medicine specialist in Ethiopia. Repeated remarks of facilitators and academic leaders encouraged a commitment to research and scholarly writing related to Family Medicine. The closing session provided a list of action steps that could further develop the new specialty.

Evaluations from this CME workshop demonstrated that there is significant interest in developing the specialty within Ethiopia. The design and implementation of the workshop took place with strong support from the Ethiopian Medical Association as well as the Faculty of Medicine at Addis Ababa University.

## CONCLUSION

A CME workshop can be used effectively to educate physicians about the specialty of Family Medicine. Workshop participants can learn from a global survey of the history and contributions of Family Medicine and interactive educational methods can be used to extrapolate a list of roles, skills and values that could characterise a Family Medicine specialist in a particular context.

With burgeoning interest in the specialty of Family Medicine across sub-Saharan Africa, there will be an ongoing need for creative and practical ways to introduce policymakers, academics and medical trainees to the specialty. This CME workshop proved to be an enjoyable and effective way to educate a diverse group of physicians about the nature and benefits of Family Medicine. The workshop successfully advanced the dialogue about how the specialty could take shape and the action steps that need to be taken.
